# Genome Degradation in *Brucella ovis* Corresponds with Narrowing of Its Host Range and Tissue Tropism

**DOI:** 10.1371/journal.pone.0005519

**Published:** 2009-05-13

**Authors:** Renee M. Tsolis, Rekha Seshadri, Renato L. Santos, Felix J. Sangari, Juan M. García Lobo, Maarten F. de Jong, Qinghu Ren, Garry Myers, Lauren M. Brinkac, William C. Nelson, Robert T. DeBoy, Samuel Angiuoli, Hoda Khouri, George Dimitrov, Jeffrey R. Robinson, Stephanie Mulligan, Richard L. Walker, Philip E. Elzer, Karl A. Hassan, Ian T. Paulsen

**Affiliations:** 1 Medical Microbiology and Immunology, University of California Davis, Davis, California, United States of America; 2 J. Craig Venter Institute, La Jolla, California, United States of America; 3 Escola de Veteranaria, Universidade Federal de Minas Gerais, Belo Horizonte, Brazil; 4 Molecular Biology Department, University of Cantabria, Santander, Spain; 5 California Animal Health and Food Safety Laboratory, Davis, California, United States of America; 6 Department of Veterinary Science, Louisiana State University, Baton Rouge, Louisiana, United States of America; 7 Department of Chemistry and Biomolecular Sciences, Macquarie University, Sydney, Australia; University of Hyderabad, India

## Abstract

*Brucella ovis* is a veterinary pathogen associated with epididymitis in sheep. Despite its genetic similarity to the zoonotic pathogens *B. abortus*, *B. melitensis* and *B. suis*, *B. ovis* does not cause zoonotic disease. Genomic analysis of the type strain ATCC25840 revealed a high percentage of pseudogenes and increased numbers of transposable elements compared to the zoonotic *Brucella* species, suggesting that genome degradation has occurred concomitant with narrowing of the host range of *B. ovis*. The absence of genomic island 2, encoding functions required for lipopolysaccharide biosynthesis, as well as inactivation of genes encoding urease, nutrient uptake and utilization, and outer membrane proteins may be factors contributing to the avirulence of *B. ovis* for humans. A 26.5 kb region of *B. ovis* ATCC25840 Chromosome II was absent from all the sequenced human pathogenic *Brucella* genomes, but was present in all of 17 *B. ovis* isolates tested and in three *B. ceti* isolates, suggesting that this DNA region may be of use for differentiating *B. ovis* from other Brucella spp. This is the first genomic analysis of a non-zoonotic *Brucella* species. The results suggest that inactivation of genes involved in nutrient acquisition and utilization, cell envelope structure and urease may have played a role in narrowing of the tissue tropism and host range of *B. ovis*.

## Introduction

Although previous studies have supported the notion of *Brucella* as a monospecies genus with different biotypes [1], it is still largely accepted that the genus *Brucella* is divided into six species, named according to their preferential hosts. Each one of the species is host-adapted, but not host-restricted [2]–[4]. Four of the six *Brucella* species, namely *B. melitensis*, *B. abortus*, *B. suis*, and *B. canis*, are capable of causing human disease. *B. melitensis* and *B. suis* are the most pathogenic, whereas *B. abortus* is considered of moderate pathogenicity, and *B. canis* is considered of low pathogenicity for humans. There are no reports of human infections with *B. ovis* or *B. neotomae* [3]. In addition to the six classical *Brucella* species, *Brucella* has also been isolated from marine mammals, and two species, *B. pinnipedialis* and *B. ceti* have been proposed [5]. *Brucella* isolates from marine mammals can cause human infections, with one reported case of infection due to laboratory exposure [6], and two reported cases of natural infections resulting in neurological disease [7].

*Brucella ovis* was initially recognized in the beginning of the 1950's in New Zealand and Australia as a bacterial agent associated with epididymitis and abortion in sheep [8]. Since then this organism has been isolated in several countries [9], and is considered one of the most important causes of ovine infertility, with a significant economic impact on the sheep industry [10]. *B. ovis* has a worldwide distribution in areas where sheep are economically significant, with the exception of the Great Britain [9]. The prevalence in herds ranges from 9.1 to 46.7% [11], and the seroprevalence within positive herds varies between 2.1 to 67% [11]–[14].

*B. ovis* is stably rough, and it is one of the two classical *Brucella* species that do not have zoonotic potential. In sheep, the organism causes either clinical or sub-clinical chronic infections characterized by epididymitis, orchitis, male infertility, and occasionally abortion in pregnant ewes [15]. Sexually mature rams are more susceptible than young males [16]. However, *B. ovis* infection may affect males as young as 4 months old [9]. Natural transmission apparently occurs through mucosal membranes, and venereal transmission is significant when a female previously mated with an infected male copulates with a second susceptible male during the same period of estrus [17]. Upon invasion through mucosal membranes, *B. ovis* initially resides in local lymph nodes, which is followed by bacteremia and finally colonization of the genital tract around 30 days post infection [18]. The factors defining the genital tropism of this organism remain unknown.

Sequencing of *B. melitensis*, and *B. suis* genomes demonstrated a high level of similarity between the two genomes, with over 90% of the genes having more than 98–100% nucleotide identity [19]. Furthermore, comparison between these two species resulted in the identification of only 32 and 43 genes that were unique to *B. melitensis* and *B. suis*, respectively. This level of variability is remarkably low even when compared to variations between serotypes within the same bacterial species such as in serotypes Typhi and Typhimurium of *Samonella enterica* [20]. More recently, the complete genome sequence of *B. abortus* (strains 9–941 and 2308) became available confirming the striking similarity both among different species of *Brucella* and within the species *B. abortus* [21], [22]. Comparisons between these three *Brucella* species revealed more than 94% identity at the nucleotide level. In addition, comparisons between the genomes of the two *B. abortus* strains that have been sequenced (9–941 and 2308) resulted in identification of only a small number of strain-specific deletions and polymorphisms [21]. The genetic similarity among *Brucella* species has been confirmed by whole genome hybridizations [23]. Together these studies support the original hybridization studies performed more than 20 years ago suggesting that *Brucella* is a monospecific genus from the genetic point of view [1]. Considering the high level of identity among *Brucella* species pathogenic to humans, the comparison of those species with a *Brucella* lacking the potential to cause human infections may bring new insights into host specificity and pathogenesis. In this study we present the genome analysis of the rough non-human-pathogen *Brucella ovis* and compare its genome with those of the zoonotic *Brucella* spp. Our analysis focused on two sets of genomic features (i) deletions and gene degradation that could potentially result in loss of virulence factors important for infection of hosts other than sheep, and (ii) *B. ovis*-specific genes that could contribute to genital tract tropism or cause a reduction in virulence for non-ovine hosts.

## Results and Discussion

### General features of the *B. ovis* genome

**Figure 1:**
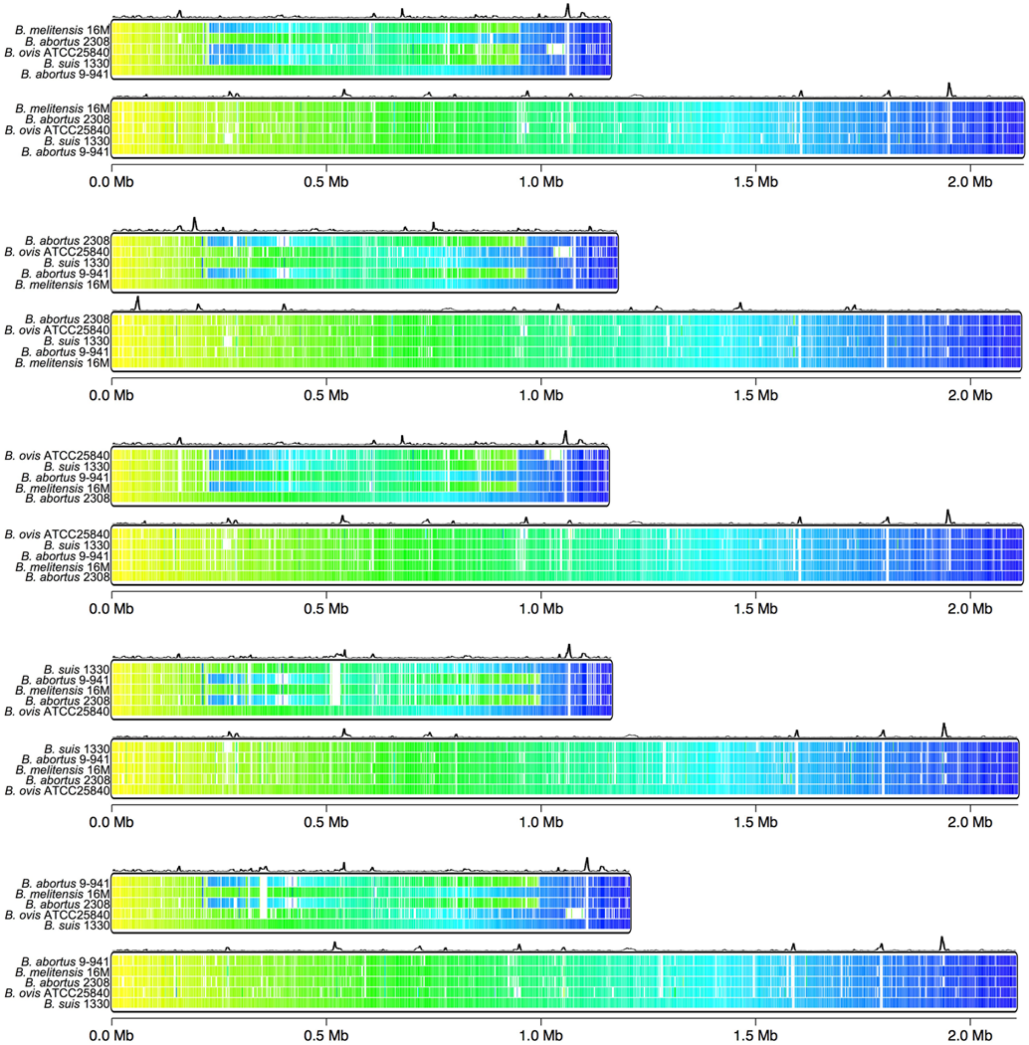
Whole genome alignment of sequenced *Brucella* strains. The five genomes were compared to each other by using COG, BLAST and HMM analyses. Each genome is colored with a gradient that ranges from yellow (nucleotide 1) to blue (end). Differences in color between a reference sequence (the bottom colored line in each set) and the other genomes indicate conserved protein-coding regions that have been rearranged. Uncolored segments denote coding regions in which no conserved genes were detected. The curves on top of each panel represent the nucleotide composition (Χ^2^ analysis) (see *Materials and Methods*) of the reference strain of the panel, and peaks indicate regions of atypical composition.

**Figure 2:**
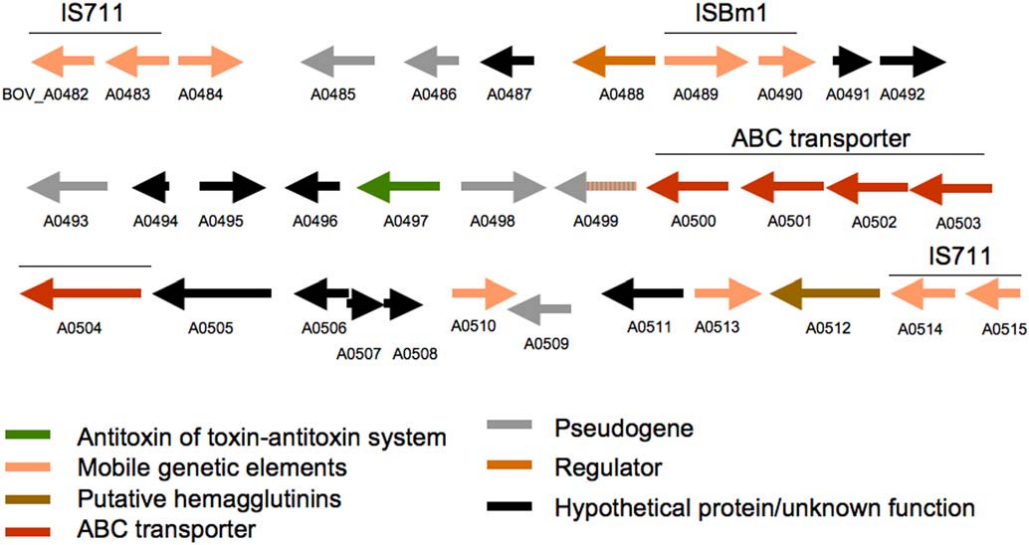
Map of the *B. ovis*-specific island on Chromosome II, which resembles a composite transposon. Genes are color-coded according to functional predictions.

The *B. ovis* type strain ATCC25840 (also known as 63/290 or NCTC10512) used for sequencing was isolated from a sheep in Australia in 1960 [8]. The genome of this strain consists of two circular chromosomes of 2,111,370 bp (Chromosome I; NCBI Accession Number NC_009505) and 1,164,220 bp (Chromosome II; NCBI Accession Number NC_009504), which are predicted to encode a total of 2890 proteins, 1928 on ChrI and 962 on ChrII (Table 1). Features of the *B. ovis* genome are summarized in Table 1. Comparison with the sequenced genomes of *B. suis* (GenBank Accession numbers NC_004310 and NC_004311) [24], *B. abortus* (GenBank Accession numbers NC_007618 and NC_007624) [21], [22] and *B. melitensis* 16M (GenBank Accession numbers NC_003317 and NC_003318) [19] shows a large degree of conservation, particularly in the % G+C content and size of the chromosomes. This comparison also revealed several species-specific differences, including regions missing from *B. ovis* relative to the other sequenced *Brucella* genomes and genes unique to *B. ovis*. These differences are listed in Table 1 and Figures 1–2, and are discussed below.

**Table 1:** Characteristics of the *B. ovis* ATCC 25840 genome

	*B. ovis* ATCC 25840		*B. suis* 1330^1^		*B. abortus* 2308^1^		*B. melitensis* 16 M^2^	
	Chr I	Chr II	Chr I	Chr II	Chr I	Chr II	Chr I	Chr II
**Size (bp)**	2,111,370	1,164,220	2,107,792	1,207,381	2,121,359	1,156,950	2,117,144	1,177,787
**G+C content (%)**	57.2	57.2	57.2	57.3	57.2	57.3	57.2	57.3
**No. of protein-coding genes**	1,928	962	2,123	1,149	2,000	1,034	2,090	1,075
**No. of rRNA operons**	2	1	2	1	2	1	2	1
**No. of tRNAs**	39	14	41	14	41	14	40	14
**No. of pseudogenes**	119	125	61	1	186	130	83	80
**% pseudogenes**	5.8	11.5	2.8	0.1	8.5	11.2	3.8	6.9
**IS*711* copies**	25	13	5	2	5	2	5	2

1 obtained from NCBI

2 obtained from the PATRIC database

### Comparison to *B. suis*, *B. abortus*, and *B. melitensis* proteomes

There is a significant degree of overlap between the predicted proteomes of sequenced *Brucella* species. Comparative best-match blastp searches identified 2282 annotated proteins in *B. ovis* that are shared with *B. suis*, *B. melitensis* and *B. abortus*, 79% of the annotated proteome. Nonetheless, this analysis suggested that the *B. ovis* genome lacks 675, 610 and 539 protein coding genes annotated in *B. suis*, *B. melitensis* and *B. abortus* 2308, respectively (Table S3). To determine whether these genes are truly absent from the *B. ovis* genome they were compared to the *B. ovis* chromosomal sequences using blastn searches. Interestingly, good matches were found for 64, 125 and 18 genes thought to be lacking in *B. ovis* in the genomes of *B. suis*, *B. melitensis* and *B. abortus* 2308, respectively (Table S3). There are several possible reasons for these genes not being highlighted as shared using comparative best-match blastp searches, e.g., they may be pseudogenes in *B. ovis* and therefore, not part of the predicted proteome, they may be the products of duplications where only one duplication product is matched in best match searches or differences in gene annotation between the strains resulted in these sequences not being annotated as a protein coding gene in *B. ovis*. In total only 33 annotated protein coding genes in *B. ovis* are unique to this species.

### Genomic rearrangements

Gradient figures were used to compare the chromosomes of *B. ovis* with those of the other sequenced *Brucella* genomes. No large inversions or rearrangements were observed in the *B. ovis* genome compared to the sequenced genomes of *B. suis* or *B. melitensis* (Figure 1).

### Genomic deletions

#### Chromosome I

Four deletions of 4 kb or larger were identified in the *B. ovis* genome, compared to its closest relative, *B. suis*. Chromosome I lacks a 15 kb region that encompasses *B. suis BR0966-BR0987*. Interestingly, in *B. suis*, this region is inserted into the sequence of tRNAgly and is adjacent to a phage integrase-like gene, features suggestive of horizontal gene transfer. This region contains the *wboA* glycosyl transferase gene, shown to be essential for lipopolysaccharide biosynthesis [25], and a second glycosyl transferase, as well as several hypothetical proteins. The lack of the two glycosyl transferases likely contributes to the rough LPS phenotype of *B. ovis*. These findings were in agreement with previous reports by Vizcaino et al and Rajashekara et al [23], [26] showing the absence of these genes from *B. ovis*.

A second deletion on Chromosome I of 7745 bp encompasses *BR1078-BR1083*. This island in *B. suis* contains three hypothetical genes and two site specific recombinases and is flanked by two tRNA-Leu genes, of which one remains in *B. ovis*. A smaller deletion of 3954 bp on Chromosome I has led to loss of two genes (*BR1852-BR1853*) encoding a transcriptional regulator and a protein predicted to be a branched chain amino acid permease. This deletion is also associated with transposable elements, as these two genes in *B. suis* are flanked by copies of IS*2020* [27], one of which remains in *B. ovis*.

#### Chromosome II

The 44.5 kb island in *B. suis* (*BRA1074-BRA1113*; [28]), containing four predicted ABC transport systems and three transcriptional regulators, is absent from the *B. ovis* genome. The presence of two copies of IS*1239* flanking two pseudogenes in *B. ovis* suggests that this is the result of a loss of this region by recombination.

The 18.3 kb IncP island on *B. suis* Chromosome II containing *BRA0362-BRA0379*, previously reported to be present in *B. suis* biovars 1–4, *B. canis, B. neotomae*, and marine mammal isolates but missing in *B. melitensis* [24], [28], is also absent from the *B. ovis* genome, as reported by Lavigne and colleagues [28].

### Unique genomic regions

Chromosome II, contains an island of 26.5 kb (*BOV_A0482-BOV_A0515*) with structure suggestive of a composite transposon (Fig. 2). This island likely overlaps the *B. ovis*-specific 21-kb SpeI fragment of the small chromosome identified previously by genome mapping [29]. Proteins encoded on the island include transposases, an ABC transporter, a putative hemagglutinin, and several hypothetical proteins. This region is present in 17/17 *B. ovis* strains tested, suggesting a high level of conservation within the species (Supplementary Table S2). Interestingly, the predicted product of *BOV_A0497* exhibits similarity to antitoxins of toxin-antitoxin maintenance systems [30], suggesting a possible selective pressure for maintenance of this genetic island in *B. ovis*. Genome sequence data and analysis of the island by PCR showed that this region is absent from the genomes of *B. suis*, *B. abortus*, *B. melitensis*, *B. canis* and *B. neotomae* (Table 2), suggesting initially that it may be specific to *B. ovis*. However, we detected this island in three marine isolates of *Brucella* from captive bottlenose dolphins [31], [32]. The protein coding genes within this unique region constitute the majority of the unique protein coding genes in *B. ovis* (Supplementary Table S3).

**Table 2:** Detection of the *B. ovis* genomic island in *Brucella* species

	Species									
ORF	*Brucella ovis*	*B. melitensis*	*B. abortus*	*B. suis*	*B. canis*	*B. neotomae*	*B. ceti* (Muu)	*B. ceti* (Cudo)	*B. ceti* (Nor)	NTC^1^
A0492	+	−	−	−	−	−	+	+	+	−
A0495	+	−	−	−	−	−	+	+	+	−
A0496	+	−	−	−	−	−	+	+	+	−
A0497	+	−	−	−	−	−	+	+	+	−
A0500	+	−	−	−	−	−	+	+	+	−
A0502	+	−	−	−	−	−	+	+	+	−
A0503	+	−	−	−	−	−	+	+	+	−
A0504	+	−	−	−	−	−	+	+	+	−
A0505	+	−	−	−	−	−	+	+	+	−
A0506	+	−	−	−	−	−	+	+	+	−
A0511	+	−	−	−	−	−	+	+	+	−
A0512	+	−	−	−	−	−	+	+	+	−
BR0615^2^	+	+	+	+	+	+	+	+	+	−

1 NTC, no template control

2 Positive control for amplification reaction

### Transposable elements

The genome of *B. ovis* contains 38 complete copies of IS*711*, confirming previous estimates obtained by hybridization [33]. 25 of the copies are located in Chromosome I, and 13 in Chromosome II. Interestingly, several of these copies appear to be expanded clonally, suggesting that they could be active in *B. ovis*. In 17 cases, IS*711* is inserted in copies of a repeated sequence of the BruRS family [34]. Five of the IS*711* copies appear to disrupt genes, which could be a contributing factor to the general process of genome degradation observed in *B. ovis*. A case of special interest is the *B. ovis*-specific island (see above), where a 25 kb region between two copies of IS*711* in *B. ovis* is absent in all the other sequenced species, suggestive of either deletion by recombination between the two copies in the other *Brucella* species, or of acquisition of the region with duplication of IS*711*.

### Pseudogenes

It has been proposed that the unique complement of pseudogenes in each of the *Brucella* species may contribute to their differing degrees of infectivity and host preference [21], [22]. Interestingly *B. abortus*, which is less virulent for humans than *B. suis* and *B. melitensis*, has a greater number of pseudogenes (316) than *B. suis* 1330 (62) or *B. melitensis* 16M (163) (Table 1). Similar to *B. abortus*, the *B. ovis* genome contains a large number of pseudogenes with 244 identified. The small chromosome contains more pseudogenes (125; 11.3%) than the large chromosome (119; 5.7%) (Table 1). Of the 244 *B. ovis* pseudogenes, 40 are hypothetical or conserved hypothetical genes, 62 have predicted transport functions, 23 are defective transposases, and 14 are predicted to have regulatory functions. The finding that some pseudogenes in the *B. melitensis* flagellar region encode full-length proteins raises the question of whether some pseudogenes in *B. ovis* may also be functional [35].

### Metabolism

#### Urease

Based on biotyping, *B. ovis* is urease-negative. However it contains the two urease clusters described in all the other sequenced *Brucella* genomes. Urease has been reported to be important for the ability of *B. abortus* [36] and *B. suis* [37] to survive passage through the stomach in the mouse model of infection. The importance of urease for oral infection is consistent with a lack of human infections observed with *B. ovis*, despite identification of this organism in the milk of infected ewes [38]. The *ure1* cluster is the only one showing urease activity at least in *B. abortus, B. melitensis,* and *B. suis* [36], [37], while the *ure2* does not have any demonstrable urease activity. The *ure1* cluster of *B. ovis* contains several point mutations that are predicted to result in conserved changes shared by at least one other urease positive species. However, UreC1 contains a 30 bp deletion that would cause a loss of 10 amino acids in UreC1. Although all the residues described to be important for enzymatic activity of UreC1 are conserved [39], this deletion must render the urease inactive. Moreover, complementation with the *ureC1* gene from *B. melitensis* (Sangari, unpublished results) results in urease activity. Regarding the *ure2* cluster, *ureF2* (*BOV_1316*) and *ureT* (*BOV_1319*) are pseudogenes, while the remaining genes are conserved, suggesting that this cluster could encode an unknown activity.

#### Sugar metabolism

*B. ovis* is defective in oxidative metabolism of arabinose, galactose, ribose, xylose, glucose and erythritol [40]. Analysis of the genome sequence reveals a possible basis for these metabolic defects in *B. ovis* relative to the human pathogenic *Brucella* species. Several putative sugar transporters predicted to be functional in other *Brucella* species appear to be inactivated by frameshifts, point mutations or gene degradation in *B. ovis*. Further, *pckA* encoding phosphoenolpyruvate carboxykinase (*BOV_2009*) is inactivated by frameshift, which would affect the gluconeogenesis pathway and the ability of *B. ovis* to utilize pyruvate, amino acids, or glycerol as carbon sources.

#### Erythritol

Erythritol is the preferred carbon source of *B. abortus* [41], [42]. Erythritol is metabolized in *Brucella* by the enzymes encoded in the *eryABCD* operon[43] Moreover, it has been recently described that the carbohydrate transport system located upstream of the *ery* operon constitutes the erythritol transport system in *Rhizobium leguminosarum* bv. *Viciae* [44], and that the operon immediately downstream also forms part of the erythritol catabolic pathway. Microarray experiments have revealed that these three operons are regulated by erythritol in *B. abortus*, reinforcing that the three clusters participate in erythritol catabolism (Sangari et al, unpublished). *B. ovis* does not oxidize erythritol, and it is not inhibited by its presence in the growth media. This is reflected in the genome by the presence of a stop codon in *eryA* (*BOV_A0811*) and a frameshift in *eryD* (*BOV_A0814*) that render them pseudogenes. In addition two genes of the putative ABC erythritol transport system, *eryF* and *eryG* (BOV_A805 and BOV_A806)) carry mutations that render them pseudogenes (a 2 bp deletion resulting in an premature stop codon). The mutation in *eryD* could have an effect in the over expression of all genes of the *ery* system. On the contrary, mutations in the transport genes and in *eryA* block the entry of erythritol in the cell and its phosphorylation, avoiding the accumulation of toxic intermediates and the depletion of ATP observed in the vaccine strain S19 [45]. The accumulation of mutations in these two clusters suggest that they may no longer be needed by *B. ovis*. The third cluster is intact, and the enzymes use substrates that are central (or core) carbohydrate metabolites such as dihydroxyacetone phosphate, glyceraldehydes-3-phosphate, and other three carbon compounds that can be produced after decarboxylation of erythritol and its derivatives.

While it has been hypothesized that erythritol may serve as a carbon source during growth of *B. abortus*, *B. suis*, and *B. melitensis* in the placenta of their natural hosts, an analysis of deletion mutants in these models has not been reported. However, the lower incidence of abortion in sheep flocks infected with *B. ovis* compared to *B. melitensis* correlates with the inability of *B. ovis* to use erythritol as a carbon source. Mutants in *eryB* and *eryC* have been described to have a limited ability to grow in macrophages [46]. This limitation may well contribute to the limited virulence of *B. ovis*.

#### Glucose and Galactose

Unlike *B. melitensis*, *B. abortus* and *B. suis*, *B. ovis* is unable to grow on glucose or galactose as a primary carbon source [40]. Analysis of genes involved in uptake and utilization of these carbon sources reveals that *B. ovis gluP* (*BOV_A0172*), encoding a glucose/galactose transporter [47] is a pseudogene. *B. abortus gluP* mutants have a reduced ability to persist in the spleens of mice [48], suggesting that the ability to utilize these carbon sources may be important for systemic persistence of the human pathogenic *Brucella* species. However *B. suis* also lacks GluP and is able to oxidize glucose and galactose, suggesting that additional functions are lacking in *B. ovis* that contribute to utilization of these carbon sources. Several predicted sugar ABC transporters (*BOV_1299-BOV1301, BOV_A0278-BOV_A0282*, *BOV_A0645-BOV_A0648* and *BOV_A1083-BOV_A1086*) of unknown specificity contain pseudogenes, which may potentially reduce the ability of *B. ovis* to take up glucose and galactose.

#### Ribose

Two components, a permease and an ATP binding protein of a putative ribose ABC transport system (*BOV_A0936-BOV_A0937*) are pseudogenes in *B. ovis*, suggesting that the inability of *B. ovis* to utilize ribose as a sole carbon source may be the result of a reduced capacity to take up ribose from the growth medium. Similarly, a periplasmic binding protein and an ATP binding protein of a predicted ABC transporter for xylose (*BOV_A1055-BOVA1056*) contain frameshifts, which may underlie the inability of *B. ovis* to utilize xylose [40].

#### Oxidase phenotype

*B. ovis* is the only *Brucella* species with a negative oxidase phenotype. Oxidase phenotype depends on the activity of cytochrome C oxidase which in *B. suis* is encoded by at least 7 genes, four of them organized in one operon, *BR0363-BR0360* (*ccoNOQP*), together with *BR0467* (*coxB*), encoding cytochrome c oxidase, subunit II, BR0468 (*coxA)* encoding cytochrome c oxidase, subunit I and *BR0472* (*coxC*) encoding cytochrome c oxidase, subunit III. In *B. ovis*, the operon *ccoNOQP* (*BOV_0376-BOV_0379*), encoding the cb type cytochrome c oxidase is well conserved, except the gene *ccoO* (*BOV_0378*), which contains a frameshift generated by deletion of an A near its 5′ end, is a pseudogene. The *B. ovis coxB* gene (*BOV_0473*) contains a frameshift that very probably inactivates the gene, while *coxA* (*BOV_0474*) differs only in two residues with the *B. suis* product. The last 6 amino acids of *coxC (BOV_0478)* are lost as result of a short deletion (56 bp) that fuses this product with the next, apparently unrelated Orf. A short repeated sequence (GGGGCGGC) at both ends of the deletion seems to be responsible for this rearrangement. These genomic differences may be responsible for the oxidase negative phenotype of *B. ovis*.

#### Nitrogen metabolism

Several genes encoded in the *B. suis* genome with inferred functions in nitrogen metabolism are not predicted to be functional in the *B. ovis* genome. The genes *norB* (*BOV_A0225*), encoding the large subunit of nitric oxide reductase, and *nosX* (*BOV_A0256*), a gene of unknown function in the operon encoding nitric oxide synthase, are pseudogenes in *B. ovis*. The third gene, *narK* (*BOV_A0276*), encoding a nitrite extrusion protein, is degenerate, as was found in the *B. melitensis* and *B. abortus* 2308 genomes [21]. Nitric oxide reductase is required for survival and persistence of *B. suis* in mice [49], suggesting that lack of this activity in *B. ovis* may contribute to its restricted tissue tropism and host range.

A locus that contributes to growth of *E. coli* on aspartate as a nitrogen source, xanthine dehydrogenase (*BOV_0365-BOV_0367;* [50]) contains a pseudogene in *B. ovis*, suggesting a further defect in nitrogen metabolism. Further, this locus could function in salvage pathways for purine nucleotides, which could contribute to nitrogen metabolism. A correlation between a defective purine nucleotide salvage pathway and reduced ability of *B. ovis* to survive intracellularly would be consistent with the importance of purine biosynthetic pathways for full virulence of *B. abortus* and *B. melitensis* [51], [52]. Further, a homolog of *Sinorhizobium meliloti fixI* (*BOV_0373*), encoding an cation pump involved in symbiotic nitrogen fixation [53], is inactivated by a point mutation in the *B. ovis* genome. As the function of the *fixGHI* genes in *Brucella spp.* has not been determined, the biological significance of this gene inactivation for *B. ovis* is currently unknown.

### Host-pathogen interactions

#### Lipopolysaccharide

*B. ovis* is known to have a rough LPS phenotype, which given the critical role of O-antigen in pathogenicity of *B. abortus, B. suis* and *B. melitensis*, likely contributes to its reduced pathogenicity for laboratory animals compared to other *Brucella* spp. [8], [54], [55]. As mentioned above, the *wboA* gene is missing from *B. ovis,* as well as a second, genetically linked glycosyltransferase. Pseudogenes with a possible function in LPS biosynthesis include a glycosyltransferase (*BOV_A0475*), an LpxA family acetyltransferase (*BOV_A0367*) and a putative undecaprenylphosphate alpha-N-acetylglucosamine transferase (*BOV_A0371*) of which the latter may also be involved in murein biosynthesis. While it is known that *B. ovis* LPS has a higher affinity for antimicrobial peptides than that of *B. abortus*, it is unknown whether differences in LPS structure compared to smooth *Brucella* species affect the interaction of *B. ovis* LPS with toll-like receptors of the innate immune system [56], [57].

#### Type IV secretion system (T4SS)

The T4SS, encoded by *virB1-virB12*, is an essential virulence factor in *B. abortus*, *B. suis*, and *B. melitensis* [48], [58]–[60]. The genes *virB1-VirB12* are intact in the *B. ovis* genome, suggesting that this system is functional. Further, the gene encoding its transcriptional activator VjbR (*BOVA_0110;* [61]) is conserved. These findings are consistent with the detection of VirB5 and VirB8 expression in *B. ovis* [62]. Two co-regulated genes identified as encoding T4SS substrates, *vceA* (*BOV_1577*) and *vceC* (BOV_1003) are present in *B. ovis*, however it is currently unknown whether there may be additional T4SS effectors in other *Brucella spp.* that are absent from *B. ovis* [63]. A recent report showing that *B. ovis* strain PA is able to replicate in macrophages and HeLa cells at a rate similar to that of *B. abortus* suggests that its T4SS is functional [64].

#### Autotransporter proteins

Four predicted autotransporters have been identified in the sequenced *Brucella* genomes. Each of the sequenced *Brucella* genomes is predicted to encode a different combination of autotransporters [21], suggesting that none of these four proteins is essential for virulence, but that different combinations of autotransporters may contribute to observed differences in tissue tropism and host preference. The *B. ovis* genome is predicted to encode three functional autotransporters, corresponding to *B. suis BR0072*, *BRA0173* and *BRA1148*, (*BOV_0071*, *BOV_A0152* and *BOV_A1053*) while the fourth gene, *BOV_1937*, corresponding to *BR2013*, is degenerate. These proteins show a range in similarity (at the amino acid level) to their *B. suis* counterparts, from 99% between *BRA1148* and *BOV_A1053*, to 86% similarity between BOV_A0152 and *BRA0173*. *BR2013*, designated *omaA,* is a pseudogene in both *B. abortus* and *B. melitensis*, and was noted to have a polymorphism with unknown functional effects in the genome of vaccine strain *B. abortus* 19 [65]. Since the product of this gene has been shown to contribute to persistence of *B. suis* in mice [66], it is possible that lack of a functional OmaA autotransporter in *B. ovis* may contribute to its limited tissue tropism and host range. However, the finding that all four autotransporter genes are predicted to be pseudogenes in *B. melitensis*, shows that they are not absolutely required for infectivity and transmission.

#### Immunomodulatory functions

In addition to the T4SS, several genes implicated in modulation of the immune response by *Brucella* spp., are well-conserved in *B. ovis* ATCC25840. These include *btp1,* encoding the TIR domain protein, an inhibitor of TLR2 signaling, the B cell mitogen encoded by *prpA*, and cyclic β-glucan synthase [67]–[70].

#### Outer membrane proteins

The two component regulator BvrR/BvrS, which has been shown to be a master regulator of many virulence-associated functions [71], [72], appears intact in *B. ovis*. However, a putative *envZ* osmosensor (*BOV_A0412*) is a pseudogene. Two genes shown to encode outer membrane proteins in other *Brucella* species *omp2a* (*BOV_0632* [73]) and *omp31* (*BOV_1565*; [74]), contain point mutations in *B. ovis*. For Omp31, the point mutation is predicted to lead to a truncation in the protein. It was found previously that the outer membrane of *B. ovis* is more susceptible to cationic peptides than a rough *B. abortus* mutant [56], suggesting that together with the defects in LPS biosynthesis discussed above, these defects in outer membrane components may further compromise the cell envelope stability of *B. ovis* making it less able to survive environmental stresses.

### Perspective

The unique biology of *Brucella ovis* compared to the human pathogenic species appears to be in part the result of genome degradation. Worldwide, the majority of human brucellosis cases occur via ingestion of contaminated dairy products. *B. ovis* is not known to cause human infection, despite worldwide consumption of unpasteurized sheep's milk, where *B. ovis* has been detected [38]. Oral transmission, although feasible in experimental conditions, does not appear to be one of the main routes of infection for *B. ovis*, whereas passive venereal transmission via the ewe is the most important one [75]. This suggests that this species has lost the ability to infect via the oral route. One genomic change that may contribute to this loss of oral infectivity is the loss of an important virulence factor, urease, that is required for survival of stomach acidity by *Brucella* spp [36], [37]. Urease has also been shown to contribute to the establishment of *Actinobacillus pleuropneumoniae* infection in pigs through the respiratory tract [76]. If this mechanism is also operative in *Brucella* spp., then it is possible that *B. ovis* is also deficient in establishing infections not only by the digestive route but also via aerosol inhalation, which are the two main routes of infection by human pathogenic *Brucella* spp. Additional genes that seem to be non-functional in *B.* ovis could also contribute to reduce the number of transmission routes compared to other *Brucella* species. A characteristic of *B. ovis* is its tropism for the ovine male genital tract, which presents as epididymo-orchitis [8]. Since other *Brucella* species, especially *B. melitensis* are known to cause epididymo-orchitis in human patients [77], the predilection of *B. ovis* to cause epididymo-orchitis in rams likely represents a loss of functions required to target to other tissues. However, due to the large number of genes in the *B. ovis* genome with unknown function, a gain of functions that allow for increased colonization of the male genital tract cannot be ruled out based on the genome sequence.

## Materials and Methods

### Genome sequencing and annotation

The complete genome sequence of *Brucella ovis* strain ATCC25840 was determined using the whole-genome shotgun method as previously described [78]. Physical and sequencing gaps were closed using a combination of primer walking, generation and sequencing of transposon-tagged libraries of large-insert clones, and multiplex PCR [79]. Identification of putative protein-encoding genes and annotation of the genome were performed as previously described [24]. An initial set of genes predicted to encode proteins was identified with GLIMMER [80]. Genes consisting of fewer than 30 codons and those containing overlaps were eliminated. Frame shifts and point mutations were corrected or designated ‘authentic.’ Functional assignment, identification of membrane-spanning domains, and determination of paralogous gene families were performed as previously described [24]. Sequence alignments and phylogenetic trees were generated using the methods described previously [24].

### Trinucleotide composition

Distribution of all 64 trinucleotides (3-mers) was determined, and the 3-mer distribution in 1,000-bp windows that overlapped by half their length (500 bp) across the genome was computed. For each window, we computed the χ^2^ statistic on the difference between its 3-mer content and that of the whole chromosome. A large value for indicates the 3-mer composition in this window is different from the rest of the chromosome (minimum of two standard deviations). Probability values for this analysis are based on assumptions that the DNA composition is relatively uniform throughout the genome, and that 3-mer composition is independent. Because these assumptions may be incorrect, we prefer to interpret high χ^2^ values as indicators of regions on the chromosome that appear unusual and demand further scrutiny.

### Comparative genomics

The *B. ovis* ATCC25840 genome was compared to the genomes of *B. suis* 1330, *B. abortus* 2308, *B. abortus* 9-941, and *B. melitensis* 16M (PATRIC), at the nucleotide level by suffix tree analysis using MUMmer [81], and the predicted *B. ovis* CDSs were compared with the gene sets from the other sequenced *Brucella* genomes by BLAST and by HMM paralogous family searches, as previously described [82].

### Analysis of the B. ovis-specific island

Genomic DNA from *B. ovis* strains and other *Brucella* species (*B. melitensis*, *B. suis*, *B. abortus*, *B. canis*, *B. neotomae*, and *B. pinnipedialis*) were subjected to PCR amplification of 12 target sequences within the *B. ovis*-specific island. PCR reactions were performed using 13 µL of a commercial PCR mix (PCR Supermix, Invitrogen, USA), 0.75 µL of a 25 µM solution of each primer (Supplementary Table S1), and 1 µL of DNA (100 to 500 ng per reaction). Cycling parameters were denaturation at 95°C for 5 minutes; 35 cycles of denaturation (95°C for 1 min seconds), annealing (55°C for 1 min), and extension (72°C for 1 min); and a final extension at 72°C for 5 min. PCR products were resolved by agarose gel electrophoresis.

### GenBank accession

The genomic sequence data are available at GenBank under accession numbers NC_009505 (Chromosome I) and NC_009504 (Chromosome II).

## Supporting Information

Table S1 PCR primers used to detect the *B. ovis*-specific island genes and product sizes.
(0.06 MB DOC)

Table S2 Presence of the *B. ovis*-specific island in a panel of *B. ovis* isolates
(0.09 MB DOC)

Table S3 Matches of B. ovis 25840 predicted genes with B. abortus 9-941, B. abortus 2308, B. melitensis 16M, and B. suis 1330. The 33 genes unique to B. ovis are at the top of the table. Genes missing from B. ovis that are present in the other genomes are given with a dashed line. Genes for which there is no annotation, but for which BLASTN detected the DNA sequence in the genome, are shown in bold type. These include differences resulting from annotation of the genomes. Note that the B. melitensis 16M locus designations are from PATRIC and do not match the locus tags from the annotation deposited at NCBI.
(0.55 MB XLS)

## References

[1] Verger J-M, Grimont F, Grimont PAD, Grayon M (1985). Brucella, a Monospecific Genus as Shown by Deoxyribonucleic Acid Hybridization.. Int J Syst Bacteriol.

[2] Boschiroli ML, Foulongne V, O'Callaghan D (2001). Brucellosis: a worldwide zoonosis.. Curr Opin Microbiol.

[3] Corbel MJ (1997). Brucellosis: An Overview.. EmergInfDis.

[4] Moreno E, Moriyon I (2002). Brucella melitensis: a nasty bug with hidden credentials for virulence.. Proc Natl Acad Sci U S A.

[5] Foster G, Osterman BS, Godfroid J, Jacques I, Cloeckaert A (2007). Brucella ceti sp. nov. and Brucella pinnipedialis sp. nov. for Brucella strains with cetaceans and seals as their preferred hosts.. Int J Syst Evol Microbiol.

[6] Brew SD, Perrett LL, Stack JA, MacMillan AP, Staunton NJ (1999). Human exposure to Brucella recovered from a sea mammal.. Vet Rec.

[7] Sohn AH, Probert WS, Glaser CA, Gupta N, Bollen AW (2003). Human neurobrucellosis with intracerebral granuloma caused by a marine mammal Brucella spp.. Emerg Infect Dis.

[8] Buddle MB (1956). Studies on Brucella ovis (n.sp.), a cause of genital disease of sheep in New Zealand and Australia.. J Hyg (Lond).

[9] Burgess GW (1982). Ovine contagious epididymitis: a review.. Vet Microbiol.

[10] Carpenter TE, Berry SL, Glenn JS (1987). Economics of Brucella ovis control in sheep: epidemiologic simulation model.. J Am Vet Med Assoc.

[11] Sergeant ES (1994). Seroprevalence of Brucella ovis infection in commercial ram flocks in the Tamworth area.. N Z Vet J.

[12] Maghalhães Neto A, Gil-Turnes C (1996). Brucelose ovina no Rio Grande do Sul.. Pesquisa Veteriinária Brasiliera.

[13] Robles CA, Uzal FA, Olaechea FV, Low C (1998). Epidemiological observations in a Corriedale flock affected by Brucella ovis.. Vet Res Commun.

[14] Torres E, Aparicio E, Quezada F, Tavera F, Güemes F (1997). Presencia de anticuerpos contra diferentes especies de Brucella en sementales ovonos jóvenes.. Veterinaria México.

[15] Ficapal A, Jordana J, Blasco J, Moriyon I (1998). Diagnosis and epidemiology of Brucella ovis infection in rams.. Small Ruminant Research.

[16] Walker RL, LeaMaster BR, Stellflug JN, Biberstein EL (1986). Association of age of ram with distribution of epididymal lesions and etiologic agent.. J Am Vet Med Assoc.

[17] Brown GM, Pietz DE, Price DA (1973). Studies on the transmission of Brucella ovis infection in rams.. Cornell Vet.

[18] Biberstein EL, McGowan B, Olander H, Kennedy PC (1964). Epididymitis in Rams. Studies on Pathogenesis.. Cornell Vet.

[19] DelVecchio VG, Kapatral V, Redkar RJ, Patra G, Mujer C (2002). The genome sequence of the facultative intracellular pathogen Brucella melitensis.. Proc Natl Acad Sci U S A.

[20] Tsolis RM (2002). Comparative genome analysis of the alpha -proteobacteria: relationships between plant and animal pathogens and host specificity.. Proc Natl Acad Sci U S A.

[21] Chain PS, Comerci DJ, Tolmasky ME, Larimer FW, Malfatti SA (2005). Whole-genome analyses of speciation events in pathogenic Brucellae.. Infect Immun.

[22] Halling SM, Peterson-Burch BD, Bricker BJ, Zuerner RL, Qing Z (2005). Completion of the genome sequence of Brucella abortus and comparison to the highly similar genomes of Brucella melitensis and Brucella suis.. J Bacteriol.

[23] Rajashekara G, Glasner JD, Glover DA, Splitter GA (2004). Comparative whole-genome hybridization reveals genomic islands in Brucella species.. J Bacteriol.

[24] Paulsen IT, Seshadri R, Nelson KE, Eisen JA, Heidelberg JF (2002). The Brucella suis genome reveals fundamental similarities between animal and plant pathogens and symbionts.. Proc Natl Acad Sci U S A.

[25] McQuiston JR, Vemulapalli R, Inzana TJ, Schurig GG, Sriranganathan N (1999). Genetic characterization of a Tn5-disrupted glycosyltransferase gene homolog in Brucella abortus and its effect on lipopolysaccharide composition and virulence.. Infect Immun.

[26] Vizcaino N, Caro-Hernandez P, Cloeckaert A, Fernandez-Lago L (2004). DNA polymorphism in the omp25/omp31 family of Brucella spp.: identification of a 1.7-kb inversion in Brucella cetaceae and of a 15.1-kb genomic island, absent from Brucella ovis, related to the synthesis of smooth lipopolysaccharide.. Microbes Infect.

[27] Halling SM, Zuerner RL (2002). Evidence for lateral transfer to Brucellae: characterization of a locus with a Tn-like element (Tn2020).. Biochim Biophys Acta.

[28] Lavigne JP, Vergunst AC, Bourg G, O'Callaghan D (2005). The IncP island in the genome of Brucella suis 1330 was acquired by site-specific integration.. Infect Immun.

[29] Michaux-Charachon S, Bourg G, Jumas-Bilak E, Guigue-Talet P, Allardet-Servent A (1997). Genome structure and phylogeny in the genus Brucella.. J Bacteriol.

[30] Hayes CS, Sauer RT (2003). Toxin-antitoxin pairs in bacteria: killers or stress regulators?. Cell.

[31] Ewalt DR, Payeur JB, Martin BM, Cummins DR, Miller WG (1994). Characteristics of a Brucella species from a bottlenose dolphin (Tursiops truncatus).. J Vet Diagn Invest.

[32] Miller WG, Adams LG, Ficht TA, Cheville NF, Payeur JP (1999). Brucella-induced abortions and infection in bottlenose dolphins (Tursiops truncatus).. J Zoo Wildl Med.

[33] Ouahrani S, Michaux S, Sri Widada J, Bourg G, Tournebize R (1993). Identification and sequence analysis of IS6501, an insertion sequence in Brucella spp.: relationship between genomic structure and the number of IS6501 copies.. Journal of General Microbiology.

[34] Halling SM, Bricker BJ (1994). Characterization and occurrence of two repeated palindromic DNA elements of Brucella spp.: Bru-RS1 and Bru-RS2.. Mol Microbiol.

[35] Fretin D, Fauconnier A, Kohler S, Halling S, Leonard S (2005). The sheathed flagellum of Brucella melitensis is involved in persistence in a murine model of infection.. Cell Microbiol.

[36] Sangari FJ, Seoane A, Rodriguez MC, Aguero J, Garcia Lobo JM (2007). Characterization of the urease operon of Brucella abortus and assessment of its role in virulence of the bacterium.. Infect Immun.

[37] Bandara AB, Contreras A, Contreras-Rodriguez A, Martins AM, Dobrean V (2007). Brucella suis urease encoded by ure1 but not ure2 is necessary for intestinal infection of BALB/c mice.. BMC Microbiol.

[38] Grillo MJ, Marin CM, Barberan M, Blasco JM (1999). Experimental Brucella ovis infection in pregnant ewes.. Vet Rec.

[39] Jabri E, Carr MB, Hausinger RP, Karplus PA (1995). The crystal structure of urease from Klebsiella aerogenes.. Science.

[40] Meyer ME (1969). Phenotypic comparison of Brucella ovis to the DNA-homologous Brucella species.. Am J Vet Res.

[41] Anderson JD, Smith H (1965). The Metabolism of Erythritol by Brucella Abortus.. J Gen Microbiol.

[42] Smith H, Williams AE, Pearce JH, Keppie J, Harris-Smith PW (1962). Foetal erythritol: a cause of the localization of Brucella abortus in bovine contagious abortion.. Nature.

[43] Sangari FJ, Aguero J, Garcia-Lobo JM (2000). The genes for erythritol catabolism are organized as an inducible operon in Brucella abortus.. Microbiology.

[44] Yost CK, Rath AM, Noel TC, Hynes MF (2006). Characterization of genes involved in erythritol catabolism in Rhizobium leguminosarum bv. viciae.. Microbiology.

[45] Sperry JF, Robertson DC (1975). Inhibition of growth by erythritol catabolism in Brucella abortus.. J Bacteriol.

[46] Kohler S, Foulongne V, Ouahrani-Bettache S, Bourg G, Teyssier J (2002). The analysis of the intramacrophagic virulome of Brucella suis deciphers the environment encountered by the pathogen inside the macrophage host cell.. Proc Natl Acad Sci U S A.

[47] Essenberg RC, Candler C, Nida SK (1997). Brucella abortus strain 2308 putative glucose and galactose transporter gene: cloning and characterization.. Microbiology.

[48] Hong PC, Tsolis RM, Ficht TA (2000). Identification of genes required for chronic persistence of Brucella abortus in mice.. Infect Immun.

[49] Loisel-Meyer S, Jimenez de Bagues MP, Basseres E, Dornand J, Kohler S (2006). Requirement of norD for Brucella suis virulence in a murine model of in vitro and in vivo infection.. Infect Immun.

[50] Xi H, Schneider BL, Reitzer L (2000). Purine catabolism in Escherichia coli and function of xanthine dehydrogenase in purine salvage.. J Bacteriol.

[51] Alcantara RB, Read RD, Valderas MW, Brown TD, Roop RM, 2nd (2004). Intact purine biosynthesis pathways are required for wild-type virulence of Brucella abortus 2308 in the BALB/c mouse model.. Infect Immun.

[52] Izadjoo MJ, Bhattacharjee AK, Paranavitana CM, Hadfield TL, Hoover DL (2004). Oral vaccination with Brucella melitensis WR201 protects mice against intranasal challenge with virulent Brucella melitensis 16M.. Infect Immun.

[53] Kahn D, David M, Domergue O, Daveran ML, Ghai J (1989). Rhizobium meliloti fixGHI sequence predicts involvement of a specific cation pump in symbiotic nitrogen fixation.. J Bacteriol.

[54] Allen CA, Adams LG, Ficht TA (1998). Transposon-derived Brucella abortus rough mutants are attenuated and exhibit reduced intracellular survival.. Infect Immun.

[55] Godfroid F, Taminiau B, Danese I, Denoel P, Tibor A (1998). Identification of the perosamine synthetase gene of Brucella melitensis 16M and involvement of lipopolysaccharide O side chain in Brucella survival in mice and in macrophages.. Infect Immun.

[56] Freer E, Pizarro-Cerda J, Weintraub A, Bengoechea JA, Moriyon I (1999). The outer membrane of Brucella ovis shows increased permeability to hydrophobic probes and is more susceptible to cationic peptides than are the outer membranes of mutant rough Brucella abortus strains.. Infect Immun.

[57] Lapaque N, Takeuchi O, Corrales F, Akira S, Moriyon I (2006). Differential inductions of TNF-alpha and IGTP, IIGP by structurally diverse classic and non-classic lipopolysaccharides.. Cell Microbiol.

[58] Delrue RM, Martinez-Lorenzo M, Lestrate P, Danese I, Bielarz V (2001). Identification of Brucella spp. genes involved in intracellular trafficking.. Cell Microbiol.

[59] O'Callaghan D, Cazevieille C, Allardet-Servent A, Boschiroli ML, Bourg G (1999). A homologue of the Agrobacterium tumefaciens VirB and Bordetella pertussis Ptl type IV secretion systems is essential for intracellular survival of Brucella suis.. Mol Microbiol.

[60] Sieira R, Comerci DJ, Sanchez DO, Ugalde RA (2000). A homologue of an operon required for DNA transfer in Agrobacterium is required in Brucella abortus for virulence and intracellular multiplication.. J Bacteriol.

[61] Delrue RM, Deschamps C, Leonard S, Nijskens C, Danese I (2005). A quorum-sensing regulator controls expression of both the type IV secretion system and the flagellar apparatus of Brucella melitensis.. Cell Microbiol.

[62] Rouot B, Alvarez-Martinez MT, Marius C, Menanteau P, Guilloteau L (2003). Production of the type IV secretion system differs among Brucella species as revealed with VirB5- and VirB8-specific antisera.. Infect Immun.

[63] de Jong MF, Sun YH, den Hartigh AB, van Dijl JM, Tsolis RM (2008). Identification of VceA and VceC, two members of the VjbR regulon that are translocated into macrophages by the Brucella type IV secretion system.. Mol Microbiol.

[64] Martin-Martin AI, Caro-Hernandez P, Orduna A, Vizcaino N, Fernandez-Lago L (2008). Importance of the Omp25/Omp31 family in the internalization and intracellular replication of virulent B. ovis in murine macrophages and HeLa cells.. Microbes Infect.

[65] Crasta OR, Folkerts O, Fei Z, Mane SP, Evans C (2008). Genome sequence of Brucella abortus vaccine strain S19 compared to virulent strains yields candidate virulence genes.. PLoS ONE.

[66] Bandara AB, Sriranganathan N, Schurig GG, Boyle SM (2005). Putative outer membrane autotransporter protein influences survival of Brucella suis in BALB/c mice.. Vet Microbiol.

[67] Arellano-Reynoso B, Lapaque N, Salcedo S, Briones G, Ciocchini AE (2005). Cyclic beta-1,2-glucan is a Brucella virulence factor required for intracellular survival.. Nat Immunol.

[68] Briones G, Inon de Iannino N, Roset M, Vigliocco A, Paulo PS (2001). Brucella abortus cyclic beta-1,2-glucan mutants have reduced virulence in mice and are defective in intracellular replication in HeLa cells.. Infect Immun.

[69] Salcedo SP, Marchesini MI, Lelouard H, Fugier E, Jolly G (2008). Brucella control of dendritic cell maturation is dependent on the TIR-containing protein Btp1.. PLoS Pathog.

[70] Spera JM, Ugalde JE, Mucci J, Comerci DJ, Ugalde RA (2006). A B lymphocyte mitogen is a Brucella abortus virulence factor required for persistent infection.. Proc Natl Acad Sci U S A.

[71] Guzman-Verri C, Manterola L, Sola-Landa A, Parra A, Cloeckaert A (2002). The two-component system BvrR/BvrS essential for Brucella abortus virulence regulates the expression of outer membrane proteins with counterparts in members of the Rhizobiaceae.. Proc Natl Acad Sci U S A.

[72] Sola-Landa A, Pizarro-Cerda J, Grillo MJ, Moreno E, Moriyon I (1998). A two-component regulatory system playing a critical role in plant pathogens and endosymbionts is present in Brucella abortus and controls cell invasion and virulence.. Mol Microbiol.

[73] Marquis H, Ficht TA (1993). The omp2 gene locus of Brucella abortus encodes two homologous outer membrane proteins with properties characteristic of bacterial porins.. Infect Immun.

[74] Vizcaino N, Cloeckaert A, Zygmunt MS, Dubray G (1996). Cloning, nucleotide sequence, and expression of the Brucella melitensis omp31 gene coding for an immunogenic major outer membrane protein.. Infect Immun.

[75] Blasco JM, Nielsen K, Duncan JR (1990). Brucella ovis.. Animal Brucellosis.

[76] Bosse JT, MacInnes JI (2000). Urease activity may contribute to the ability of Actinobacillus pleuropneumoniae to establish infection.. Can J Vet Res.

[77] Madkour MM, Al-Otabi KE, Al-Ahmary S, Al-Sabaan F, Al-Wahhabi B, Madkour MM (2001). Genitourinary Brucellosis.. Madkour's Brucellosis.

[78] Fraser CM, Casjens S, Huang WM, Sutton GG, Clayton R (1997). Genomic sequence of a Lyme disease spirochaete, Borrelia burgdorferi.. Nature.

[79] Tettelin H, Radune D, Kasif S, Khouri H, Salzberg SL (1999). Optimized multiplex PCR: efficiently closing a whole-genome shotgun sequencing project.. Genomics.

[80] Delcher AL, Bratke KA, Powers EC, Salzberg SL (2007). Identifying bacterial genes and endosymbiont DNA with Glimmer.. Bioinformatics.

[81] Delcher AL, Phillippy A, Carlton J, Salzberg SL (2002). Fast algorithms for large-scale genome alignment and comparison.. Nucleic Acids Res.

[82] Myers GS, Parker D, Al-Hasani K, Kennan RM, Seemann T (2007). Genome sequence and identification of candidate vaccine antigens from the animal pathogen Dichelobacter nodosus.. Nat Biotechnol.

