# Supplementary material for: Genome Degradation in Brucella ovis Corresponds with Narrowing of Its Host Range and Tissue Tropism
Source: PLoS One. 2009 May 13;4(5):e5519. doi: 10.1371/journal.pone.0005519 (PMC2677664; doi:10.1371/journal.pone.0005519)
Supplement: Table S1 — PCR primers used to detect the B. ovis-specific island genes and product sizes. (0.06 MB DOC) [file pone.0005519.s001.doc]

**Table S1:** PCR primers used to detect the *B. ovis*-specific island genes and product sizes.

| Target | Primers | Product size |
| --- | --- | --- |
| A0492 | F: 5’-TTGAGACCGCTATCGTTGAGGG-3’ R: 5’-GTCCTTGTGGTAGATGGGCAGTAG-3’ | 278 bp |
| A0495 | F: 5’-CAGGATTATGTGCTCAACGATGC-3’ R: 5’-CAGGTGCTCCAGAAACGATACC-3’ | 135 bp |
| A0496 | F: 5’-TGGCTATTACGACGACACTGGAAG-3’ R: 5’-AAGCATCACAAAGCGGGTTCGG-3’ | 278 bp |
| A0497 | F: 5’-TCATTACCAAGAACGGTCGCC-3’ R: 5’-CGCAAGATCGTCTTCTGTCAGC-3’ | 119 bp |
| A0500 | F: 5’-TGGTATCTTCAGCCGTTCCAAG-3’ R: 5’-ATCTTTGCCCGTTCCAGTCG-3’ | 225 bp |
| A0502 | F: 5’-TGATAAATGCTGAACTGCCGC-3’ R: 5’-CGCCGAAATACCAAAACAGAGG-3’ | 167 bp |
| A0503 | F: 5’-GCCTACGCTGAAACTTGCTTTTG-3’ R: 5’-ATCCCCCCATCACCATAACCGAAG-3’ | 228 bp |
| A0504 | F: 5’-TGATTGGGACGCAGACCAGAAC-3’ R: 5’-CGGGTGTAACTCCAGATTATGTCG-3’ | 159 bp |
| A0505 | F: 5’-ACCAAACCAAACTTGATGTCGG-3’ R: 5’-GCATTTTCGGCAACCATTCC-3’ | 220 bp |
| A0506 | F: 5’-GCACCAAATATGCGGAAGATCC-3’ R: 5’-TTCGATCATCGTCTGGCTCG-3’ | 140 bp |
| A0511 | F: 5’-ATGCGGCGACCAATGAACTG-3’ R: 5’-TGATGCGTTGAGGGCAATCC-3’ | 197 bp |
| A0512 | F: 5’-TTCAGGCGACTGCTAATGGCAC-3’ R: 5’-AAACCGATACCTCATCCCCGAG-3’ | 135 bp |
| BR0615 (positive control) | F: 5’- AGTCCGGAGCCTATAAGC-3’ R: 5’-AGGCTGTAGATTGCAATC-3’ | 631 bp |
