# Supplementary material for: Genome Degradation in Brucella ovis Corresponds with Narrowing of Its Host Range and Tissue Tropism
Source: PLoS One. 2009 May 13;4(5):e5519. doi: 10.1371/journal.pone.0005519 (PMC2677664; doi:10.1371/journal.pone.0005519)
Supplement: Table S2 — Presence of the B. ovis-specific island in a panel of B. ovis isolates (0.09 MB DOC) [file pone.0005519.s002.doc]

**Table S2:** Presence of the *B. ovis*-specific island in a panel of *B. ovis* isolates

|  | *B. ovis* isolates | | | | | | | | | | | | | | | | | | |
| --- | --- | --- | --- | --- | --- | --- | --- | --- | --- | --- | --- | --- | --- | --- | --- | --- | --- | --- | --- |
| ORF | ATCC 25840 | VR808 | Y732 | 14  A12 | 14  E41 | 14  E48 | Ames  IA | LSU  32 | LSU  57 | LSU  67 | LSU  68 | LSU  71 | LSU  73 | LSU  76 | LSU  82 | LSU  84 | LSU  91 | LSU  99 | NTC^1^ |
| A0492 | + | + | + | + | + | + | + | + | + | + | + | + | + | + | + | + | + | + | - |
| A0495 | + | + | + | + | + | + | + | + | + | + | + | + | + | + | + | + | + | + | - |
| A0496 | + | + | + | + | + | + | + | + | + | + | + | + | + | + | + | + | + | + | - |
| A0497 | + | + | + | + | + | + | + | + | + | + | + | + | + | + | + | + | + | + | - |
| A0500 | + | + | + | + | + | + | + | + | + | + | + | + | + | + | + | + | + | + | - |
| A0502 | + | + | + | + | + | + | + | + | + | + | + | + | + | + | + | + | + | + | - |
| A0503 | + | + | + | + | + | + | + | + | + | + | + | + | + | + | + | + | + | + | - |
| A0504 | + | + | + | + | + | + | + | + | + | + | + | + | + | + | + | + | + | + | - |
| A0505 | + | + | + | + | + | + | + | + | + | + | + | + | + | + | + | + | + | + | - |
| A0506 | + | + | + | + | + | + | + | + | + | + | + | + | + | + | + | + | + | + | - |
| A0511 | + | + | + | + | + | + | + | + | + | + | + | + | + | + | + | + | + | + | - |
| A0512 | + | + | + | + | + | + | + | + | + | + | + | + | + | + | + | + | + | + | - |

^1^NTC, no template control
